# {2-[(2,5-Dimethyl­phen­yl)imino­methyl]pyridine-κ^2^ *N*,*N*′}diiodidozinc(II)

**DOI:** 10.1107/S1600536808020230

**Published:** 2008-08-23

**Authors:** Mohamad Reza Talei Bavil Olyai, Saeed Dehghanpour, Bita Hoormehr, Fahimeh Gholami, Hamid Reza Khavasi

**Affiliations:** a Department of Chemistry, Islamic Azad University, South Tehran Branch, Tehran, Iran; b Department of Chemistry, Alzahra University, Vanak, Tehran, Iran; c Department of Chemistry, Islamic Azad University, Karaj Branch, Tehran, Iran; d Department of Chemistry, Shahid Beheshti University, Evin, Tehran 1983963113, Iran

## Abstract

In the mol­ecule of the title compound, [ZnI_2_(C_14_H_14_N_2_)], the Zn atom is four-coordinated in a distorted tetra­hedral geometry by two N atoms of the Schiff base ligand and by two I atoms. The benzene and pyridine rings are oriented at a dihedral angle of 70.75 (3)°. The five-membered ring has an envelope conformation. There is a weak π–π inter­action between benzene rings, with a centroid-to-centroid distance of 3.975 (4) Å.

## Related literature

For general background, see: Gibson *et al.* (2007 ▶); Ittel *et al.* (2000 ▶); Gibson & Spitzmesser (2003 ▶); Bart *et al.* (2004 ▶); Sugiyama *et al.* (2004 ▶); Kooistra *et al.* (2004 ▶); Bouwkamp *et al.* (2006 ▶). For related literature, see: Dehghanpour *et al.* (2007 ▶).
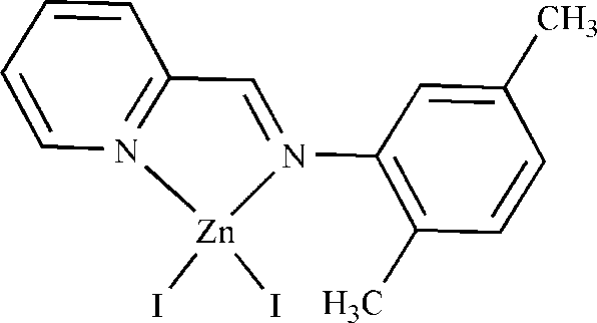


## Experimental

### 

#### Crystal data

[ZnI_2_(C_14_H_14_N_2_)]
*M* *_r_* = 529.46
Monoclinic, 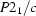

*a* = 11.467 (5) Å
*b* = 9.627 (4) Å
*c* = 15.868 (6) Å
β = 103.88 (3)°
*V* = 1700.6 (12) Å^3^
*Z* = 4
Mo *K*α radiation
μ = 5.06 mm^−1^
*T* = 298 (2) K
0.5 × 0.4 × 0.25 mm

#### Data collection

Stoe IPDSII diffractometer
Absorption correction: numerical [*X-RED32* and *X-SHAPE* (Stoe & Cie, 2005 ▶)] *T*_min_ = 0.100, *T*_max_ = 0.280
10947 measured reflections
4498 independent reflections
4035 reflections with *I* > 2σ(*I*)
*R*_int_ = 0.098

#### Refinement

*R*[*F* ^2^ > 2σ(*F* ^2^)] = 0.071
*wR*(*F* ^2^) = 0.230
*S* = 1.12
4498 reflections
172 parameters
H-atom parameters constrained
Δρ_max_ = 1.97 e Å^−3^
Δρ_min_ = −1.66 e Å^−3^

### 

Data collection: *X-AREA* (Stoe & Cie, 2005 ▶); cell refinement: *X-AREA*; data reduction: *X-RED32* (Stoe & Cie, 2005 ▶); program(s) used to solve structure: *SHELXS97* (Sheldrick, 2008 ▶); program(s) used to refine structure: *SHELXL97* (Sheldrick, 2008 ▶); molecular graphics: *ORTEP-3 for Windows* (Farrugia, 1997 ▶); software used to prepare material for publication: *WinGX* (Farrugia, 1999 ▶).

## Supplementary Material

Crystal structure: contains datablocks global, I. DOI: 10.1107/S1600536808020230/hk2486sup1.cif

Structure factors: contains datablocks I. DOI: 10.1107/S1600536808020230/hk2486Isup2.hkl

Additional supplementary materials:  crystallographic information; 3D view; checkCIF report

## Figures and Tables

**Table:** 

Zn1—I1	2.5201 (11)
Zn1—I2	2.5517 (12)
Zn1—N1	2.059 (5)
Zn1—N2	2.104 (5)

**Table:** 

I1—Zn1—I2	120.26 (4)
N1—Zn1—N2	80.16 (19)
N1—Zn1—I1	112.45 (14)
N2—Zn1—I1	107.27 (13)
N1—Zn1—I2	107.58 (15)
N2—Zn1—I2	121.99 (13)

**Fig. 1.**
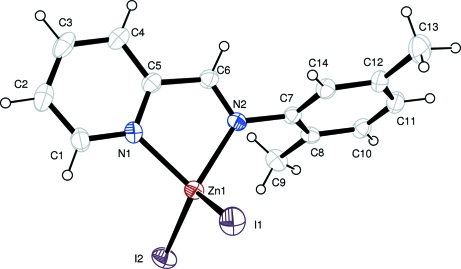
The molecular structure of the title molecule, with the atom-numbering scheme. Displacement ellipsoids are drawn at the 30% probability level.

## supplementary crystallographic information

### Comment

Transition metal compounds containing Schiff base ligands have been of great interest for many years. These compounds play an important role in the development of coordination chemistry (Gibson *et al.*, 2007). Aryl-substituted iminopyridine complexes have emerged as a powerful class of catalysts for a host of important bond-forming reactions including olefin polymerization (Ittel *et al.*, 2000; Gibson & Spitzmesser, 2003), hydrogenation and hydrosilation (Bart *et al.*, 2004). It is also now well established that iminopyridines are both redox (Sugiyama *et al.*, 2004) and chemically active ligands (Kooistra *et al.*, 2004) participating in electron transfer, addition reactions and deprotonation chemistry (Bouwkamp *et al.*, 2006). We report herein the crystal structure of the title compound.

In the molecule of the title compound (Fig. 1), the Zn atom is four-coordinated in distorted tetrahedral geometry (Table 1) by two N atoms of the Schiff base ligand, and two I atoms. The bond angles around the Zn atom deviate from the ideal tetrahedral geometry (Dehghanpour *et al.*, 2007). Rings A (N1/C1–C5) and B (C7/C8/C10/C11/C12/C14) are, of course, planar, and they are oriented at a dihedral angle of 70.75 (3)°. Ring C (Zn1/N1/N2/C5/C6) has envelope conformation, with N2 atom displaced by 0.104 (3) Å from the plane of the other ring atoms. The weak π–π interaction between B rings CgB···CgB^i^ [symmetry code: (iv) 1 - x, 1 - y, 1 - z] may stabilize the structure, with a centroid–centroid distance of 3.975 (4) Å.

### Experimental

For the preparation of the title compound, (2,5-dimethyl-N-phenyl)(pyridine-2-yl)methanimine (21.0 mg, 0.1 mmol), and ZnI_2_ (31.9 mg, 0.1 mmol) were dissolved in acetonitrile (50 ml). The mixture was stirred for 10 min at room temperature. The resulting solution was left in air for a few days, giving yellow crystals of the title compound (yield; 79%). Calc.: C 31.75 H 2.67, N 5.29%, found: C 31.79, H 2.49, N 5.31%.

### Refinement

H atoms were positioned geometrically, with C—H = 0.93 and 0.96 Å for aromatic and methyl H, and constrained to ride on their parent atoms, with U_iso_(H) = 1.2U_eq_(C).

### Crystal data

**Table:**

[ZnI_2_(C_14_H_14_N_2_)]	*F*_000_ = 992
*M**_r_* = 529.46	*D*_x_ = 2.068 Mg m^−^^3^
Monoclinic, *P*2_1_/*c*	Mo *K*α radiation λ = 0.71073 Å
Hall symbol: -P 2ybc	Cell parameters from 2112 reflections
*a* = 11.467 (5) Å	θ = 2.5–29.4º
*b* = 9.627 (4) Å	µ = 5.06 mm^−^^1^
*c* = 15.868 (6) Å	*T* = 298 (2) K
β = 103.88 (3)º	Block, yellow
*V* = 1700.6 (12) Å^3^	0.5 × 0.4 × 0.25 mm
*Z* = 4	

### Data collection

**Table:**

Stoe IPDSII diffractometer	*R*_int_ = 0.098
rotation method scans	θ_max_ = 29.4º
Absorption correction: numerical[X-RED and X-SHAPE (Stoe & Cie, 2005)]	θ_min_ = 2.5º
*T*_min_ = 0.100, *T*_max_ = 0.280	*h* = −15→11
10947 measured reflections	*k* = −11→13
4498 independent reflections	*l* = −21→21
4035 reflections with *I* > 2σ(*I*)	

### Refinement

**Table:**

Refinement on *F*^2^	H-atom parameters constrained
Least-squares matrix: full	*w* = 1/[σ^2^(*F*_o_^2^) + (0.1439*P*)^2^ + 2.419*P*] where *P* = (*F*_o_^2^ + 2*F*_c_^2^)/3
*R*[*F*^2^ > 2σ(*F*^2^)] = 0.071	(Δ/σ)_max_ = 0.007
*wR*(*F*^2^) = 0.230	Δρ_max_ = 1.97 e Å^−^^3^
*S* = 1.12	Δρ_min_ = −1.66 e Å^−^^3^
4498 reflections	Extinction correction: none
172 parameters	

### Special details

**Table:**

Geometry. All e.s.d.'s (except the e.s.d. in the dihedral angle between two l.s. planes) are estimated using the full covariance matrix. The cell e.s.d.'s are taken into account individually in the estimation of e.s.d.'s in distances, angles and torsion angles; correlations between e.s.d.'s in cell parameters are only used when they are defined by crystal symmetry. An approximate (isotropic) treatment of cell e.s.d.'s is used for estimating e.s.d.'s involving l.s. planes.

### Fractional atomic coordinates and isotropic or equivalent isotropic displacement parameters (Å^2^)

**Table:**

	*x*	*y*	*z*	*U*_iso_*/*U*_eq_	
Zn1	0.83553 (6)	0.25679 (7)	0.02157 (4)	0.0406 (2)	
I1	0.69958 (5)	0.45620 (5)	0.03661 (4)	0.0622 (2)	
I2	0.87622 (5)	0.19974 (6)	−0.12580 (3)	0.0617 (2)	
N1	0.9989 (4)	0.2665 (5)	0.1106 (3)	0.0400 (9)	
N2	0.8103 (4)	0.1007 (5)	0.1083 (3)	0.0366 (8)	
C1	1.0919 (6)	0.3467 (9)	0.1080 (5)	0.0524 (14)	
H1	1.086	0.4083	0.062	0.063*	
C2	1.1962 (6)	0.3412 (9)	0.1712 (5)	0.0590 (18)	
H2	1.2614	0.3967	0.1681	0.071*	
C3	1.2026 (6)	0.2495 (9)	0.2411 (5)	0.0596 (19)	
H3	1.2721	0.2453	0.2856	0.072*	
C4	1.1071 (5)	0.1665 (8)	0.2440 (4)	0.0498 (13)	
H4	1.1104	0.1042	0.2893	0.06*	
C5	1.0055 (5)	0.1786 (6)	0.1771 (4)	0.0411 (11)	
C6	0.8989 (5)	0.0951 (6)	0.1752 (4)	0.0412 (11)	
H6	0.8952	0.0384	0.2219	0.049*	
C7	0.7036 (5)	0.0265 (5)	0.1120 (3)	0.0356 (9)	
C8	0.6663 (5)	−0.0814 (6)	0.0540 (3)	0.0377 (10)	
C9	0.7345 (7)	−0.1256 (8)	−0.0114 (5)	0.0531 (14)	
H9A	0.8136	−0.1554	0.0183	0.064*	
H9B	0.7404	−0.0488	−0.0487	0.064*	
H9C	0.693	−0.201	−0.0455	0.064*	
C10	0.5634 (5)	−0.1522 (6)	0.0595 (4)	0.0440 (12)	
H10	0.5352	−0.2247	0.0213	0.053*	
C11	0.5023 (5)	−0.1160 (7)	0.1214 (5)	0.0495 (13)	
H11	0.4335	−0.1652	0.1241	0.059*	
C12	0.5402 (5)	−0.0092 (8)	0.1791 (4)	0.0491 (13)	
C13	0.4725 (8)	0.0300 (13)	0.2483 (7)	0.076 (3)	
H13A	0.4453	0.1244	0.2396	0.091*	
H13B	0.5253	0.0207	0.305	0.091*	
H13C	0.4047	−0.0304	0.2435	0.091*	
C14	0.6415 (5)	0.0638 (6)	0.1732 (4)	0.0416 (11)	
H14	0.668	0.138	0.2104	0.05*	

### Atomic displacement parameters (Å^2^)

**Table:**

	*U*^11^	*U*^22^	*U*^33^	*U*^12^	*U*^13^	*U*^23^
Zn1	0.0414 (4)	0.0406 (4)	0.0392 (4)	−0.0024 (2)	0.0082 (3)	0.0058 (2)
I1	0.0607 (3)	0.0455 (3)	0.0819 (4)	0.00774 (18)	0.0200 (3)	0.0016 (2)
I2	0.0745 (4)	0.0713 (4)	0.0442 (3)	0.0013 (2)	0.0239 (2)	0.00481 (18)
N1	0.036 (2)	0.038 (2)	0.047 (2)	−0.0030 (17)	0.0124 (18)	−0.0020 (18)
N2	0.041 (2)	0.033 (2)	0.0357 (19)	0.0000 (16)	0.0107 (16)	0.0014 (16)
C1	0.048 (3)	0.060 (4)	0.052 (3)	−0.015 (3)	0.019 (3)	−0.006 (3)
C2	0.039 (3)	0.076 (5)	0.064 (4)	−0.011 (3)	0.016 (3)	−0.030 (4)
C3	0.039 (3)	0.079 (5)	0.055 (4)	0.001 (3)	0.001 (3)	−0.032 (4)
C4	0.040 (3)	0.054 (3)	0.050 (3)	0.003 (2)	0.001 (2)	−0.006 (3)
C5	0.041 (2)	0.040 (3)	0.041 (2)	0.000 (2)	0.006 (2)	−0.007 (2)
C6	0.040 (2)	0.037 (2)	0.044 (3)	0.0001 (19)	0.005 (2)	0.006 (2)
C7	0.036 (2)	0.031 (2)	0.037 (2)	0.0000 (17)	0.0042 (17)	0.0039 (17)
C8	0.045 (2)	0.033 (2)	0.034 (2)	0.0013 (19)	0.0083 (19)	0.0027 (18)
C9	0.066 (4)	0.048 (3)	0.050 (3)	−0.003 (3)	0.023 (3)	−0.011 (3)
C10	0.040 (2)	0.036 (2)	0.051 (3)	−0.005 (2)	0.002 (2)	0.001 (2)
C11	0.037 (2)	0.050 (3)	0.060 (3)	−0.002 (2)	0.008 (2)	0.006 (3)
C12	0.033 (2)	0.064 (4)	0.051 (3)	0.002 (2)	0.010 (2)	0.001 (3)
C13	0.054 (4)	0.105 (7)	0.077 (5)	0.010 (4)	0.031 (4)	−0.010 (5)
C14	0.042 (3)	0.042 (3)	0.040 (2)	−0.002 (2)	0.008 (2)	−0.006 (2)

### Geometric parameters (Å, °)

**Table:**

Zn1—I1	2.5201 (11)	C7—C8	1.387 (7)
Zn1—I2	2.5517 (12)	C7—N2	1.430 (7)
Zn1—N1	2.059 (5)	C8—C10	1.384 (8)
Zn1—N2	2.104 (5)	C8—C9	1.502 (9)
C1—N1	1.326 (8)	C9—H9A	0.96
C1—C2	1.365 (10)	C9—H9B	0.96
C1—H1	0.93	C9—H9C	0.96
C2—C3	1.406 (13)	C10—C11	1.381 (10)
C2—H2	0.93	C10—H10	0.93
C3—C4	1.365 (11)	C11—C12	1.377 (10)
C3—H3	0.93	C11—H11	0.93
C4—C5	1.380 (8)	C12—C14	1.380 (9)
C4—H4	0.93	C12—C13	1.535 (11)
C5—N1	1.341 (8)	C13—H13A	0.96
C5—C6	1.457 (8)	C13—H13B	0.96
C6—N2	1.283 (7)	C13—H13C	0.96
C6—H6	0.93	C14—H14	0.93
C7—C14	1.382 (8)		
			
I1—Zn1—I2	120.26 (4)	C14—C7—C8	122.2 (5)
N1—Zn1—N2	80.16 (19)	C14—C7—N2	119.3 (5)
N1—Zn1—I1	112.45 (14)	C8—C7—N2	118.5 (5)
N2—Zn1—I1	107.27 (13)	C10—C8—C7	117.3 (5)
N1—Zn1—I2	107.58 (15)	C10—C8—C9	119.8 (6)
N2—Zn1—I2	121.99 (13)	C7—C8—C9	122.9 (5)
C1—N1—C5	119.8 (6)	C8—C9—H9A	109.5
C1—N1—Zn1	127.7 (5)	C8—C9—H9B	109.5
C5—N1—Zn1	112.4 (4)	H9A—C9—H9B	109.5
C6—N2—C7	117.4 (5)	C8—C9—H9C	109.5
C6—N2—Zn1	111.5 (4)	H9A—C9—H9C	109.5
C7—N2—Zn1	129.6 (3)	H9B—C9—H9C	109.5
N1—C1—C2	121.7 (7)	C11—C10—C8	120.4 (6)
N1—C1—H1	119.2	C11—C10—H10	119.8
C2—C1—H1	119.2	C8—C10—H10	119.8
C1—C2—C3	118.3 (7)	C12—C11—C10	121.9 (6)
C1—C2—H2	120.8	C12—C11—H11	119
C3—C2—H2	120.8	C10—C11—H11	119
C4—C3—C2	120.2 (6)	C11—C12—C14	118.1 (6)
C4—C3—H3	119.9	C11—C12—C13	121.8 (7)
C2—C3—H3	119.9	C14—C12—C13	120.1 (7)
C3—C4—C5	117.4 (7)	C12—C13—H13A	109.5
C3—C4—H4	121.3	C12—C13—H13B	109.5
C5—C4—H4	121.3	H13A—C13—H13B	109.5
N1—C5—C4	122.5 (6)	C12—C13—H13C	109.5
N1—C5—C6	116.3 (5)	H13A—C13—H13C	109.5
C4—C5—C6	121.2 (6)	H13B—C13—H13C	109.5
N2—C6—C5	119.2 (5)	C12—C14—C7	120.0 (6)
N2—C6—H6	120.4	C12—C14—H14	120
C5—C6—H6	120.4	C7—C14—H14	120
			
N1—C1—C2—C3	−1.3 (11)	C4—C5—N1—C1	−0.7 (9)
C1—C2—C3—C4	1.4 (11)	C6—C5—N1—C1	179.5 (6)
C2—C3—C4—C5	−1.1 (10)	C4—C5—N1—Zn1	179.3 (5)
C3—C4—C5—N1	0.8 (10)	C6—C5—N1—Zn1	−0.5 (6)
C3—C4—C5—C6	−179.5 (6)	N2—Zn1—N1—C1	177.4 (6)
N1—C5—C6—N2	5.8 (8)	I1—Zn1—N1—C1	−77.9 (6)
C4—C5—C6—N2	−174.0 (6)	I2—Zn1—N1—C1	56.8 (6)
C14—C7—C8—C10	−0.2 (8)	N2—Zn1—N1—C5	−2.6 (4)
N2—C7—C8—C10	179.5 (5)	I1—Zn1—N1—C5	102.1 (4)
C14—C7—C8—C9	−178.4 (6)	I2—Zn1—N1—C5	−123.2 (4)
N2—C7—C8—C9	1.3 (8)	C5—C6—N2—C7	−174.8 (5)
C7—C8—C10—C11	−0.6 (8)	C5—C6—N2—Zn1	−7.7 (7)
C9—C8—C10—C11	177.6 (6)	C14—C7—N2—C6	62.5 (7)
C8—C10—C11—C12	0.3 (10)	C8—C7—N2—C6	−117.1 (6)
C10—C11—C12—C14	0.9 (10)	C14—C7—N2—Zn1	−101.8 (6)
C10—C11—C12—C13	−179.5 (7)	C8—C7—N2—Zn1	78.6 (6)
C11—C12—C14—C7	−1.7 (9)	N1—Zn1—N2—C6	5.6 (4)
C13—C12—C14—C7	178.7 (7)	I1—Zn1—N2—C6	−105.0 (4)
C8—C7—C14—C12	1.4 (9)	I2—Zn1—N2—C6	110.3 (4)
N2—C7—C14—C12	−178.3 (5)	N1—Zn1—N2—C7	170.6 (5)
C2—C1—N1—C5	0.9 (10)	I1—Zn1—N2—C7	60.0 (5)
C2—C1—N1—Zn1	−179.0 (5)	I2—Zn1—N2—C7	−84.7 (5)
